# Arch-Support Insoles Benefit the Archery Performance and Stability of Compound Archers

**DOI:** 10.3390/ijerph19148424

**Published:** 2022-07-10

**Authors:** Ting-Ting Wu, Shin-Liang Lo, Hui Chen, Jeng-Sheng Yang, Hsien-Te Peng

**Affiliations:** 1 Graduate Institute of Coaching Science, Chinese Culture University, Taipei 11114, Taiwan; a8000631@ulive.pccu.edu.tw; 2 Department of Physical Education, Chinese Culture University, Taipei 11114, Taiwan; lxl6@faculty.pccu.edu.tw (S.-L.L.); ch2@ulive.pccu.edu.tw (H.C.); yzs2@ulive.pccu.edu.tw (J.-S.Y.)

**Keywords:** plantar pressure, balance, performance, sport biomechanics, shooting score, kinetics

## Abstract

The purpose of this study was to analyze the effects of the use of arch-support insoles on the archery performance and center of plantar pressure (CoP) excursion in compound archers. Fifteen highly skilled compound archers were the subjects. A pressure plate was used to measure the CoP excursion and percentage distribution of plantar pressure. The parameters were compared between archers wearing flat and arch-support insoles using a paired-sample *t*-test. The results demonstrated that the shooting score in archers wearing the arch-support insole was significantly greater than in those wearing the flat insoles. The CoP excursion of the left foot, right foot, and both feet in archers wearing the arch-support insole were significantly smaller than in those wearing the flat insole. The distributed percentage of the plantar pressure showed that the arch-support insole significantly reduced the plantar pressure in the left posterior zone by 3.54% compared with the flat insole, and increased the plantar pressure in the right anterior zone by 2.54%. The principal conclusion was that compound archers wearing arch-support insoles during the arrow-release process can reduce the CoP excursion of the foot and increase their shooting score. The plantar pressure was distributed evenly in arch-support insoles.

## 1. Introduction

Our feet provide standing stability and walking ability, and serve the functions of support, shock absorption, and push-off. However, they bear the entire weight of the body, and are prone to fatigue after prolonged use, which may result in pain and injury [1]. A uniform load distribution on the feet during walking or sports activities is desirable [2]. An inability to buffer the impact of ground reaction forces (GRFs) is likely to cause pain in the feet [3]. Besides muscle training to increase foot muscle strength, another method commonly adopted to reduce foot pain is the use of arch-support insoles (ASIs), which reduces the number of incorrect points of force application on the soles of the feet [1,4]. Obesity, prolonged standing, and poor foot structure are common causes of excessive plantar pressure. One of the methods used in physiotherapeutic intervention for the relief of excessive plantar pressure is the use of ASIs to maintain the foot arch within the normal range of load-bearing positions, which helps to reduce the occurrence of inflammation and pain [5]. Prolonged standing during archery practice and competitions causes a gradual increase in plantar pressure, which may lead to the development of pain. Archers may benefit from the use of ASIs.

ASIs also serve as an interventional method in many types of sports for the maintenance of good whole-body balance ability and stability in athletes to enhance sports performance. They differ from typical flat insoles (FLIs) in that they contain three-dimensional structures at the arch area for support. Previous studies on the use of ASIs in athletes have revealed that ASIs can alter lower-limb joint angles during participation in sports activities. This allows the lower-limb joints to return to normal load-bearing positions, thereby enhancing the stability of activity [6,7]. For instance, it was found that the use of ASIs in female soccer players led to an increase in heel angle and effective heightening of the medial longitudinal arch [6]. Other studies have shown that ASIs increased stability during landing and decreased the internal rotation angle in the knees of female basketballers, and reduced the ankle valgus angle during jumping in male basketballers [8,9].

In archery, good accuracy and balance stability are required to enable shot arrows to fly straight towards the target and achieve good results. To shoot an arrow, the archer has to maintain a stationary stance with feet placed shoulder width apart, turn the shoulder of the bow arm outwards and maintain it at an angle of approximately 90 degrees, hold the bow steady with the bow arm pointed towards the target, and draw the bow using the draw arm [1]. Previous archery-related research has found that excursion of the center of plantar pressure (CoP) causes a tilt in shooting posture, leading to instability in the movement of the archer. It has also been found that CoP excursion arising from anterior–posterior and medial–lateral GRFs during the shooting process is significantly smaller in highly skilled archers than in beginners [10]. A greater CoP excursion in archers indicates higher instability in the control of shooting posture. In general, archers with a higher level of expertise have a lower degree of CoP excursion, as well as better coordination and control over their shooting posture [10,11].

Past research has solely focused on observations of the effects of CoP excursion on archery techniques, with few studies investigating whether intervention with aids enhances shooting accuracy, or if ASI intervention improves the balance stability and sports performance of archers. Related studies have also mainly been conducted on recurve archers rather than compound archers [10,11]. Therefore, the present study aimed to analyze the effects of the use of ASIs on archery performance and CoP excursion in compound archers based on the hypothesis that using ASIs enhances the sports performance and stability of compound archers.

## 2. Materials and Methods

### 2.1. Subjects

In the present study, 15 compound archers were selected as subjects [11,12]. Inclusion criteria were that they were highly skilled university-level compound archers and had participated in national-level competitions. Exclusion criteria were that they had any physical illnesses or injury to the upper or lower limbs within the experimental period or 6 months prior to the experiment. They had 7.4 ± 5.9 years of experience of compound archery training and trained for 36 h a week. An a priori sample size calculation was performed using a free online tool, G*Power (www.gpower.hhu.de (accessed on 2 June 2022)), with a power level of 95% and an α level of 0.05 [13]. The expected effect size was calculated using means (0.36 and 0.67) and standard deviation (0.17 and 0.32) of the anterior–posterior COP (AP-COP) during shooting [12]. It revealed that the sample size of 11 participants would be sufficient for the analysis. All subjects used the left arm as the bow arm and the right arm as the draw arm. Signed written informed consent was obtained from all subjects, and the study was conducted in accordance with the research standards of the World Medical Association Declaration of Helsinki.

### 2.2. Procedures

**Figure 1:**
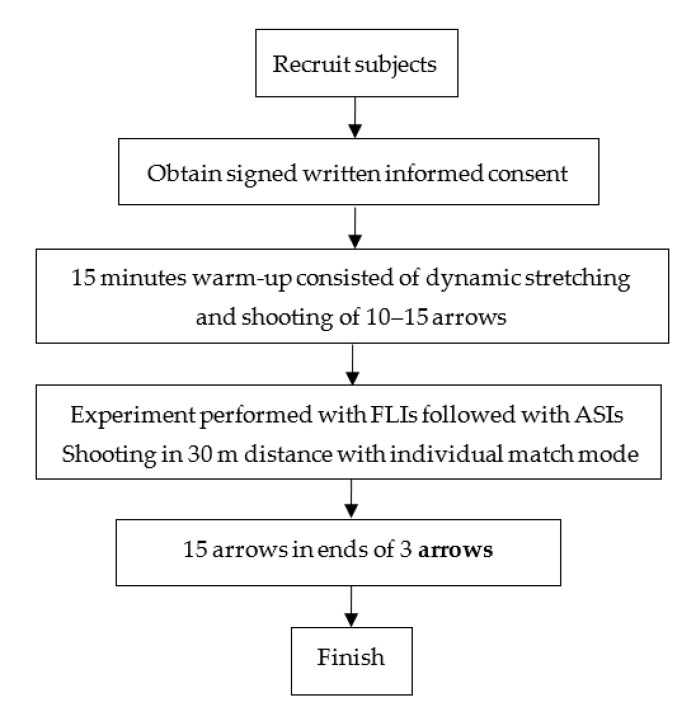
A schematic representation of the testing protocol.

Intervention was performed with FLIs followed with ASIs during the experiment, with all subjects wearing the same brand and style of footwear. Prior to the experiment, each subject performed a warm-up routine that lasted approximately 15 min and consisted of dynamic stretching and shooting of 10–15 arrows. Each subject stood on a shooting line at a distance of 30 m from the target face, and maintained the same stance while shooting each arrow during the experiment. An individual match mode was adopted for each experimental intervention, with each subject shooting 15 arrows in ends of 3 arrows. Subjects were required to complete each end within 2 min, and retrieve the shot arrows before commencing the next end. A schematic representation of the testing protocol is shown in Figure 1.

### 2.3. Instruments

During the shooting process, each subject stood on a pressure plate (Footplate, Currex GmbH, Hamburg, Germany) for collection of plantar pressure data. A round pressure sensor patch with a diameter of 1.8 cm (Switch, Delsys, Natick, MA, USA) was adhered to the anchor point on the face of each subject for the acquisition of pressure signals at the anchor point. The sampling frequency was set at 1000 Hz. The pressure plate and pressure sensor patch were synchronously activated for data acquisition.

### 2.4. Data Analysis

**Figure 2:**
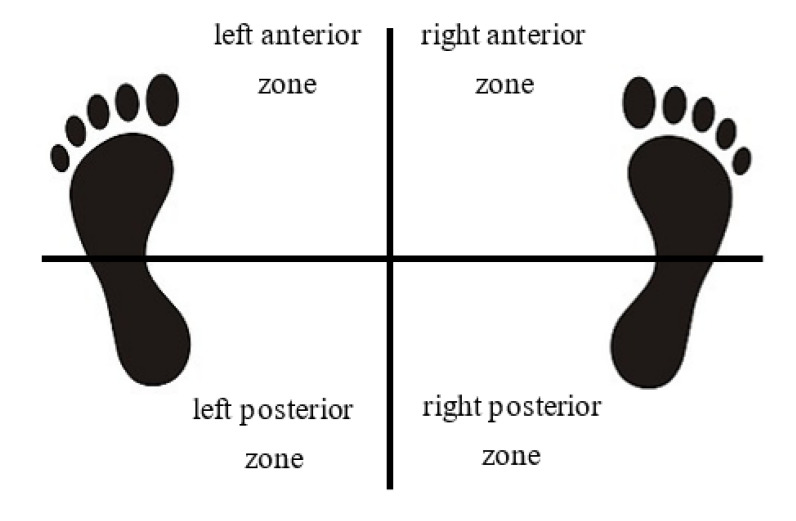
Zones of the plantar pressure distribution.

The points of impact (POIs) of the 15 arrows shot by each subject were recorded and the shooting scores were calculated. According to the rules of World Archery, to determine the shooting score, an arrow shall be scored according to the position of the shaft in the target face. For the individual compound match round, in each set, an athlete can score a maximum of 30 points (for three arrows). The athlete should shoot 5 sets and the total points are accumulated. Plantar pressure data were processed using pressure plate software (Footplate, Currex GmbH, Hamburg, Germany), and the CoP excursion and plantar pressure distribution parameters (expressed as percentages of overall pressure) at 5 s, after the appearance of a pressure signal source at the anchor point, were extracted. Excursion data included the amount of anterior–posterior and medial–lateral excursion of the CoP in the left and right foot, and in both feet. Excursion area was calculated using the anterior–posterior and medial–lateral excursion values. For each subject, the overall plantar pressure area of both feet was divided into the left anterior, left posterior, right anterior, and right posterior zones, and the percentage distribution of plantar pressure across the four zones was analyzed (Figure 2).

### 2.5. Statistics

SPSS 18.0 for Windows (SPSS Science Inc., Chicago, IL, USA) was used for the statistical analyses. A paired-sample *t*-test was performed to compare the shooting score, anterior–posterior excursion, medial–lateral excursion, and excursion area of the CoP in the left and right foot and in both feet, as well as the left anterior, left posterior, right anterior, and right posterior zones of the percentage distribution between trials with participants wearing flat versus arch-support insoles. Levene’s test was used to test the homogeneity of variance. A Kolmogorov–Smirnov test was used to evaluate the normality of the data, and a Wilcoxon test was used when the data were not normally distributed. The significance level was set at α = 0.05. The effect size (ES) for the difference between each pair of groups was calculated for each variable as a measure of the practical relevance of significance using Cohen’s d; ES values between 0.20 and 0.49 were considered small, those between 0.50 and 0.79 were considered moderate, and those of 0.80 and above were considered large [14]. It is assumed that there will be significant differences between FLIs and ASIs. 

## 3. Results

### 3.1. Shooting Score

In terms of shooting score, we found that wearing arch-support insoles significantly improved the score of arrows compared with wearing flat insoles (*p* < 0.001) (Table 1). 

### 3.2. CoP Excursion

**Figure 3:**
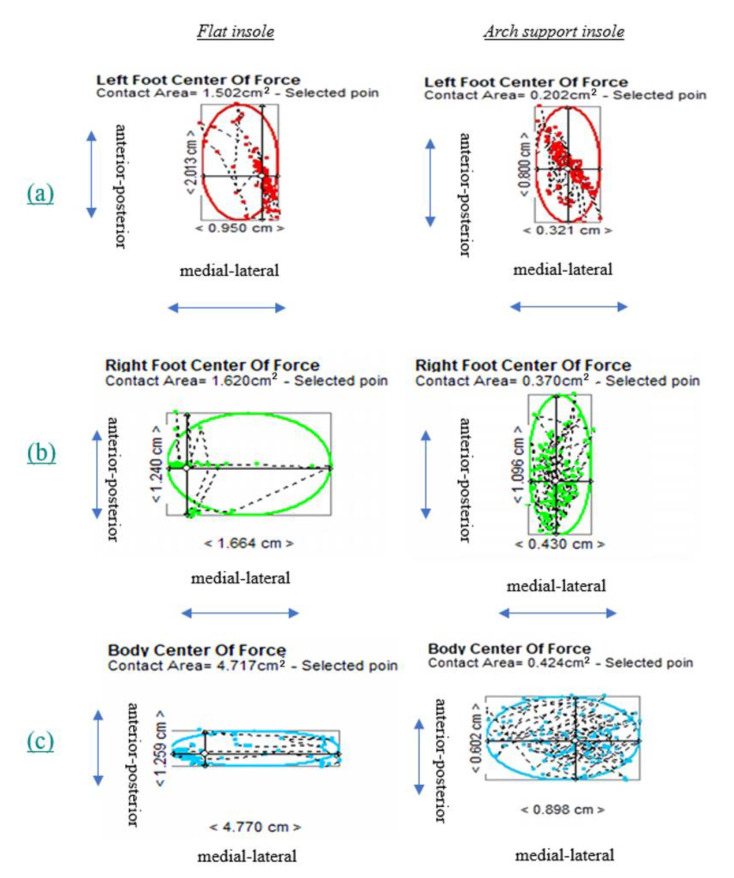
The CoP of the (**a**) left foot (red color), (**b**) right foot (green color), and (**c**) both feet (blue color) in the flat insole (**left**) and arch-support insole (**right**) of a subject. Numbers aligned with vertical axis denote anterior–posterior excursion; numbers aligned with horizontal axis denotes medial–lateral excursion.

**Figure 4:**
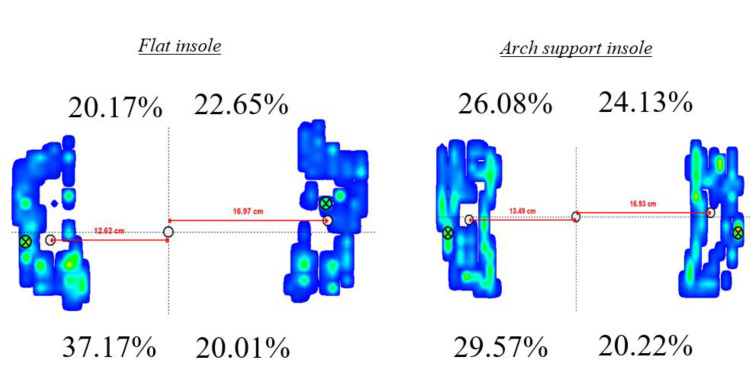
The plantar pressure distribution in the flat insole (**left**) and arch-support insole (**right**). The percentage denotes distribution of plantar pressure across the four zones.

In terms of the CoP excursion, the results showed that wearing the arch-support insole significantly reduced anterior–posterior excursion in the left foot (*p* = 0.022); significantly reduced anterior–posterior excursion (*p* = 0.000), medial–lateral excursion (*p* = 0.009) and excursion area (*p* = 0.033) in the right foot; and significantly reduced anterior–posterior excursion (*p* = 0.014), medial–lateral excursion (*p* = 0.014) in both feet. It can be seen from Figure 2, Figure 3 and Figure 4 that the sway of wearing an arch-support insole was more towards the center point than wearing a flat insole (Table 1, Figure 3).

### 3.3. Four Zones

From the percentage of plantar pressure distributed in the four zones, the results of this study showed that wearing the arch-support insole significantly reduced the plantar pressure in the left posterior region by 3.54% compared with wearing the flat insole (*p* = 0.016), and increased the plantar pressure in the right anterior region by 2.54% (*p* = 0.014) (Table 1, Figure 4). The rest of parameters did not show significant differences.

## 4. Discussion

Compared with the use of FLIs, the use of ASIs significantly increased the shooting score; decreased the anterior–posterior excursion of the CoP of the left foot, the anterior–posterior and medial–lateral excursions and excursion area of the right foot, and the overall anterior–posterior and medial–lateral excursions of both feet; and decreased the plantar pressure distribution by 3.54% in the left posterior zone and increased it by 2.54% in the right anterior zone (Table 1, Figure 4).

The use of ASIs significantly increased the anterior–posterior stability of the left foot during shooting (Figure 3). Previous studies have indicated that the stability of the bow arm is a determining factor in the arrow’s POI, and that the horizontal movement of the bow arm is generally greater than the vertical movement [15,16]. During the aiming phase, excursion or sway in the anterior–posterior and medial–lateral directions may result in low scores [11]. During the release phase, the movement trajectory in the anterior–posterior direction is affected by the posterior-to-anterior sway, with archers of a lower skill level exhibiting a greater degree of excursion and sway [10]. A greater sway of the bow arm in the horizontal direction (both feet placed parallel to the direction of the target face) may possibly be resolved by reducing the anterior–posterior excursion of the CoP of the left foot through the use of ASIs. Figure 3 indicates that the trajectories of excursion were close to the center point of the left foot. Therefore, it can be deduced that the use of ASIs enables a reduction in anterior–posterior sway, which, in turn, reduces the movement of the bow arm in the horizontal direction. During the shooting process, the left foot of the archer is responsible for supporting the torso weight and balance of the left side of the body, maintaining the stability of the bow arm, and bearing the weight of the bow. Previous research has found that the degree of bow sway is positively correlated with the direction of movement of the bow hand, with consistency between the two factors affecting the accuracy of the POIs of shot arrows [12]. Therefore, increasing the stability of the lower limbs may be beneficial to the reduction of sway in the upper limbs.

With the use of ASIs, excursion in the anterior–posterior and medial–lateral directions and excursion area of the right foot were significantly reduced, leading to an increase in stability (Table 1, Figure 3). The arrow release movement and posture are closely associated with the POI of the arrow [17]. During the draw and full draw phases of highly skilled archers who adopt a square stance, the anteroposterior direction is parallel to the target face, with movement occurring in a posterior-to-anterior direction, while the mediolateral direction is perpendicular to the target face, with movement occurring in a left-to-right direction. Greater GRFs are more experienced in the right foot than the left foot, and the sway range is smaller compared with intermediate and novice archers [10]. These results indicate that the shooting movement mostly arises from the continuous application of force towards the right side of the body. In the present study, the use of ASIs improved CoP excursion in the anterior–posterior and medial–lateral directions, as well as excursion area of the right foot of the subjects, suggesting that ASIs reduced the degree of sway in the right foot. During the shooting process, the right foot of the archer is responsible for supporting the weight of the torso and balance of the right side of the body, maintaining the stability of the upper limbs, and bearing the weight of the bowstring. Previous studies have also reported that an increase in arch contact area reduces ankle instability, thereby enhancing the overall stability of the foot [18].

Our results also demonstrated that the use of ASIs significantly decreased the overall CoP excursion and increased the overall anterior–posterior and medial–lateral stability of both feet (Table 1, Figure 3). The sway speed and area of the CoP of highly skilled archers are generally lower, and high scores have also been associated with a smaller CoP sway area [10]. Previous research has reported that a reduction in the sway speed of the body and degree of bow movement after arrow release in highly skilled archers is beneficial towards the enhancement of sports performance [12,16]. The purpose of the shooting process from the aiming phase to the release phase is to control the aiming trajectory and reduce the degree of bow sway. A study by Sarro et al. [12] reported that the degree of bow and body sway was lowest during the highest-scoring shot, with the body merely swaying slightly with bow movement. In the present study, the anterior–posterior and medial–lateral excursions of the overall CoP of both feet were reduced in the subjects wearing ASIs. During the shooting process, archers have to accustom their feet to the particularity of the shooting stance and reduce the degree of sway in their feet to enhance lower-limb stability, achieve a uniform distribution of plantar pressure across both feet, and maintain the CoP at a central location of both feet. They must also stabilize the center of gravity at their balancing point to decrease body sway [19]. Therefore, it can be deduced that the degree of sway of the lower limbs affects the stability of the upper limbs. The presence of arch support enables the control of ankle and foot movement, which contributes to the improvement of lower-limb stability [20]. ASIs can support the medial hindfoot and reduce the abduction angle of the ankle [21]. Given the existence of mutual interactions in the closed-chain movements of archery, the support provided to ankle stability by ASI intervention will also exert cascading effects on the range of motion of the knee [22]. Such mutual interactions may provide ASIs with the ability to enhance overall lower-limb stability.

An analysis of the distribution of plantar pressure across both feet revealed that plantar pressure was significantly decreased in the left posterior zone and significantly increased in the right anterior zone with the use of ASIs (Table 1, Figure 4). Under conditions of significant CoP excursions in both feet, a comparison of the excursions revealed that the CoP excursion in the right foot was greater than that in the left foot. Such a result may be attributed to the increase in coordination and balance abilities after ASI intervention, which led to an increase in the distribution of plantar pressure in the right anterior zone. This finding is similar to the results reported by Simsek et al. [10], who investigated the differences in the GRFs of archers with different levels of expertise, and observed that the anterior–posterior and medial–lateral movement trajectories of highly skilled archers were directed towards the right anterior zone. Therefore, it can be deduced that ASI intervention improves the balance ability of archers. Past research has shown that the use of ASIs in flat-footed individuals leads to the dispersion of plantar pressure through an increase in the foot contact area, which enables the recovery of foot arch functionality for the enhancement of stability and comfort [23]. A reduction in the CoP excursion and foot pronation angle of flat-footed individuals with the use of ASIs may be attributed to the increase in the postural stability of the feet [24], as the presence of support to the midfoot bones by ASIs provides stability to the ankles and promotes proprioception [25]. During the shooting process from the aiming phase to the moment of release, archers are required to maintain the highest level of stability to achieve good shooting performance. Changes in plantar pressure distribution are essential to prevent an imbalance in weight distribution and excessive pressure load in one foot [16]. A uniform distribution of plantar pressure in both feet may lead to better balance ability in the lower limbs, thereby improving shooting performance [26]. Our results indicate that ASI intervention causes changes in plantar pressure distribution, which leads to the enhancement of balance ability [27]. It is hoped that the effects of such an intervention will contribute to the attainment of equilibrium between the bow and body of the archer, which will enable arrow release in a stable and balanced state.

This study has several limitations: (1) due to the need to eliminate external factors that might interfere with the shooting process, such as the wind, sun, and rain, the shooting experiment was conducted indoors with the target placed at a distance of 30 m from the subjects; (2) a true experimental design was not employed, i.e., a control group was not established for comparison.

## 5. Conclusions

The principal conclusion was that the stability and scores of compound archers were increased with the use of ASIs. ASIs reduced anterior–posterior sway, which could, theoretically and practically, reduce the movement of the bow arm in the horizontal direction. Moreover, ASIs reduced the plantar pressure of the left posterior zone by 3.54% and increased it in the right anterior zone by 2.54%, during which highly skilled archers were directed towards the right anterior zone. Eventually, wearing ASIs improved shooting scores. Therefore, the use of ASIs by compound archers during practice and competitions is recommended for the improvement of archery performance.

## Figures and Tables

**Table 1:** Comparison of parameters between arch support and flat insole.

	Arch-Support Insole	Flat Insole	*p*	*ES*
Shooting score (point)	9.74±0.19	9.58±0.20	0.000 *	0.38
*Left foot*				
Anterior–posterior excursion (cm)	1.56±1.30	4.55±4.64	0.022 *	0.40
Medial–lateral excursion (cm)	0.74±0.82	2.32±3.91	0.134	0.27
$\mathrm{Excursion}\mathrm{area}({\mathrm{cm}}^{2}$)	9.86±7.09	9.68±6.93	0.476	0.01
*Right foot*				
Anterior–posterior excursion (cm)	1.86±1.27	3.95±2.27	0.000 *	0.49
Medial–lateral excursion (cm)	1.47±2.41	3.68±4.98	0.009 *	0.27
$\mathrm{Excursion}\mathrm{area}({\mathrm{cm}}^{2}$)	8.87±1.68	9.38±1.42	0.033 *	0.16
*Both feet*				
Anterior–posterior excursion (cm)	1.34±0.83	2.51±1.76	0.014 *	0.39
Medial–lateral excursion (cm)	2.61±3.31	6.75±7.05	0.014 *	0.35
$\mathrm{Excursion}\mathrm{area}({\mathrm{cm}}^{2}$)	6.22±6.10	6.63±6.58	0.182	0.03
*Distribution*				
Left anterior zone (%)	20.88±4.36	21.58±6.57	0.301	0.06
Left posterior zone (%)	40.18±9.00	43.72±8.75	0.016 *	0.20
Right anterior zone (%)	22.75±3.76	20.21±4.56	0.014 *	0.29
Right posterior zone (%)	16.09±6.40	14.50±8.28	0.215	0.11

* Significant difference found between the arch support and flat insole. *p* < 0.05.

## Data Availability

The data that support the findings of this study are available from the corresponding author upon reasonable request.
